# Platinum-group elements link the end-Triassic mass extinction and the Central Atlantic Magmatic Province

**DOI:** 10.1038/s41598-020-60483-8

**Published:** 2020-02-26

**Authors:** Christian Tegner, Andrea Marzoli, Iain McDonald, Nasrrddine Youbi, Sofie Lindström

**Affiliations:** 1 Centre of Earth System Petrology (ESP), Department of Geoscience, Aarhus University, Aarhus C, Denmark; 2 Department of Geoscience, University of Padova, Padova, Italy; 3 School of Earth & Ocean Sciences, Cardiff University, Cardiff, UK; 4 Department of Geology, Cadi Ayyad University, Marrakesh, Morocco; 5 Instituto Dom Luiz, Universidade de Lisboa, Lisbon, Portugal; 6 Faculty of Geology and Geography, Tomsk State University, Tomsk, Russia; 7 Geological Survey of Denmark and Greenland (GEUS), Copenhagen, Denmark

**Keywords:** Geochemistry, Geology

## Abstract

Elevated concentrations of iridium (Ir) and other platinum-group elements (PGE) have been reported in both terrestrial and marine sediments associated with the end-Triassic mass extinction (ETE) c. 201.5 million years ago. The source of the PGEs has been attributed to condensed vapor and melt from an extraterrestrial impactor or to volcanism. Here we report new PGE data for volcanic rocks of the Central Atlantic Magmatic Province (CAMP) in Morocco and show that their Pd/Ir, Pt/Ir and Pt/Rh ratios are similar to marine and terrestrial sediments at the ETE, and very different from potential impactors. Hence, we propose the PGEs provide a new temporal correlation of CAMP volcanism to the ETE, corroborating the view that mass extinctions may be caused by volcanism.

## Introduction

**Figure 1:**
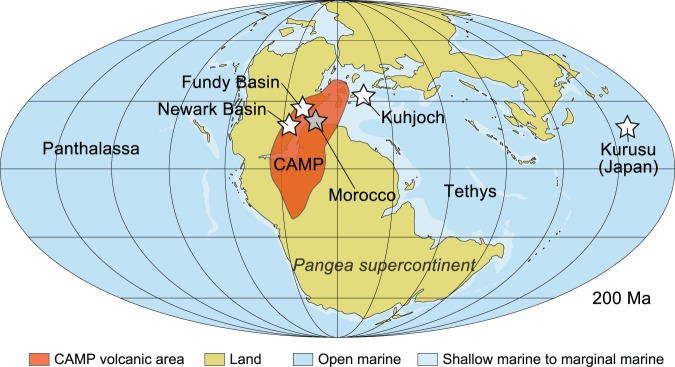
Late Triassic paleogeographic map showing the distribution of land (Pangaea supercontinent) and sea c. 200 million years ago. Also shown are: (*i*) the original extent of c. 201 million year old lava flows and intrusions of the Central Atlantic Magmatic Province (CAMP); (*ii*) the locations of a marine (Kurusu, Japan) and a continental (Fundy and Newark basins, USA) sedimentary succession of the end-Triassic mass extinction (ETE) with reported iridium (Ir) or full platinum-group element (PGE) concentrations (white stars); (*iii*) the location of the CAMP volcanics studied for PGEs in Morocco (grey star). The paleogeographic map is modified from ref. ^2^ with location of CAMP from ref. ^9^ and the locations of ETE sections with Ir and/or PGE anomalies from refs. ^16,28,31,45,49,50^.

The End Triassic Extinction event (ETE; 201.564 ± 0.022 Ma^1^) is one of the so called “Big Five” mass extinctions during the Phanerozoic era, i.e. the last 541 million years. From an ecological perspective, marine and terrestrial ecosystems were severely affected^2,3^, with estimated losses of up to 80% of all species^3,4^. Repeated and widespread magmatic activity in the Central Atlantic Magmatic Province (CAMP), a Large Igneous Province formed during the initial stages of the breakup of the Pangaea supercontinent, is often considered as the causal mechanism behind the biotic crisis^5–8^. CAMP is Earth’s largest known igneous province and covered an area larger than 10 million km^2^ on the Pangaea supercontinent (Fig. 1)^9^. The original volume of magmatic rocks is estimated to exceed 3 million km^3^ with eroded remnants of flood basalt and intrusions preserved today across Africa, Europe, North and South America^9^. The magmatic units of CAMP are mainly composed of low-Ti basaltic lavas or intrusions (sills and dykes) with distinct geochemical compositions that can be correlated across all four continents^1,9^. U-Pb chronology suggests that a large portion of the magmas were emplaced within a few hundreds of thousands of years that overlapped with the ETE^1,5,10–12^. Emissions of greenhouse gases from CAMP likely were sourced both from volcanic degassing during eruptions and thermogenic degassing from shallow intrusion of magmas into carbon-rich sedimentary basins^6,13^. Major disturbances of the carbon cycle during ETE and the succeeding Triassic–Jurassic boundary interval (201.36 ± 0.17 Ma^11^) are demonstrated by multiple negative carbon isotope excursions in δ^13^C_org_ (CIE) both in marine and continental sediments^14–20^. While it was previously thought that the lowermost preserved CAMP lavas postdated the onset of CIE’s, recent U-Pb chronology has shown that some of the intrusions are slightly older than the lavas and thus appear to coincide with or even pre-date the first CIE^5,6^. The sedimentary and fossil records preserve evidence for increased atmospheric CO_2_ interpreted as evidence for global warming^21–24^ and sites showing photic zone euxinia^25,26^ during ETE and thus contemporaneously with CAMP greenhouse gas emissions. Finally, increased concentrations of genotoxic mercury (Hg) in ETE sediments have been explained as being sourced from the CAMP volcanics^7,8,27^. Moreover, the malformation and mutagenesis of land plants (fern spores) have recently been linked to loading of volcanogenic Hg to the atmosphere^8^.

Other explanations for the causes of mass extinction associated with the ETE include bolide impact^28–30^, or a combination of bolide impact and volcanism^31^ that may have caused positive feed-back effects through destabilization of methane-hydrate reservoirs in the oceans and ocean acidification^16^. Two impact craters (Rochechouart, France^30^ and Manicouagan, Canada^32^) have previously been correlated to the end-Triassic but both have been shown to be older than ETE^33,34^. Soft-sediment deformation structures (seismites) in end-Triassic strata in the UK were originally suggested to have been impact-related^29^, but have also been mapped elsewhere across NW Europe and attributed to repeated seismicity connected to tectonic activity associated to CAMP emplacement^35,36^. Although there was an early report of shock-deformed quartz in end-Triassic sediments of Italy^32^, this has not been confirmed in subsequent studies^37,38^. Similarly, none of these authors^32,37,38^, or others, have reported spherules that are expected by a bolide impact into quartz-free oceanic crust. Thus, there remains little field or petrographic support for the impact hypothesis. The geochemical proxies, in particular Ir and the other platinum-group element (PGE) data, therefore remain the only data for which the jury is still out as to whether their origin is terrestrial or not. The impact hypothesis is, for example, convincingly demonstrated for the Cretaceous-Paleogene boundary, the youngest of the five biggest mass extinctions during the Phanerozoic era. The evidence includes not only impact spherules, shocked quartz and chronology^39–42^ but also chondritic Ir and other PGE values reported for the well-known impact layer^39,43^. Here we therefore examine new and published Ir and other PGE data associated with the end-Triassic mass extinction and CAMP volcanism, and evaluate the merits of the volcanic and impact hypotheses as the causal mechanisms of mass extinction.

## Stratigraphic framework and carbon isotope anomalies of ETE

**Figure 2:**
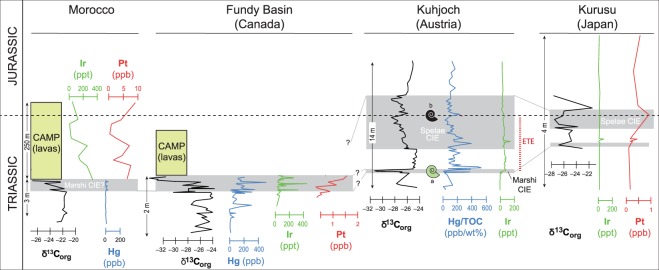
Correlation of Ir, Pt, and negative δ^13^C_org_ carbon isotope excursions (CIE) in continental sediments and CAMP lavas in the Fundy Basin and Morocco, and in marine sediments from Japan and Austria. Data from refs. ^7,14,16,18,20,28,31,45,49,50,53^. There is also a report of Ir concentrations in the Newark basin^28^ (not shown) that is very similar to Ir variations in the Fundy basin. The end-Triassic extinction interval (ETE) is located between the uppermost occurence of ammonoid *Choristoceras marshi* (marked a) and the lowermost occurence of ammonoid *Psiloceras spelae tirolicum* (marked b) and giving name to the CIE’s^44^. The thicknesses of the measured sections are shown with vertical arrows.

The stratigraphic framework for discussing the PGE data is based on Lindström *et al*.^44^ and is shown in Fig. 2. The details of the stratigraphy are best appreciated from the Global Stratotype Section and Point section for the Triassic-Jurassic boundary at Kuhjoch, Austria^18^. Here, the last occurrence of the Triassic ammonoid *Choristoceras marshi* coincides with a short and distinct negative carbon isotope excursion (CIE)^20^ (Fig. 2). This CIE is therefore denoted Marshi. Higher up in the Kuhjoch section, there is a longer negative CIE across the Triassic-Jurassic boundary that is defined by the first occurrence of the ammonoid *Psiloceras spelae tirolicum*^43,44^. This CIE is therefore denoted Spelae. The end-Triassic mass extinction (ETE) is defined as the interval between the last occurrence of the *Choristoceras marshi* ammonoid and the first occurrence of the *P. spelae* ammonoid^16,44,45^ (Fig. 2).

The correlation of the the Kujoch section to the deep marine Kurusu section in Japan, and to the continental section in the Fundy and Morocco basins is not straight forward. In the deep marine Japanese section where ammonoids are absent, the Triassic-Jurassic boundary is placed at the first occurrence of the typical lower Jurassic (Hettangian) radiolarian *Pantanellium tanuense*, which coincides with the last occurrence of conodonts, and occurs within a negative CIE which can therefore be correlated with the Spelae CIE at Kuhjoch^19,31,44,46^ (Fig. 2), and at St. Audrie’s Bay in the UK where the last conodonts are registered within the Spelae CIE. The Spelae CIE is not recorded in the continental Fundy and Morocco basins but is inferred to be correlated with CAMP magmatism. The CIEs recorded below the lowest CAMP basalts in the terrestrial basins of Fundy^47^ and Morocco^16^ are best correlated with the Marshi CIE at Kuhjoch^44^ (Fig. 2) as these sediments predate both the main extinction event based on palynological data below and above the lava flows^48^ and the Triassic-Jurassic boundary^1,11,12^.

## Iridium anomalies associated with ETE

Iridium anomalies (that serve as a proxy for other PGEs) are reported from the sedimentary successions immediately below the oldest CAMP basalts in the Newark Basin^28^ and in the Fundy Basin^49^ of NE America (Fig. 2). The Ir spike in the Newark Basin (up to 285 ppt, not shown) was recorded c. 15 metres below the base of the oldest CAMP basalt in this area, the Orange Mountain Basalt, and was interpreted as a possible signal of an extraterrestrial bolide impact, similar to the Ir anomaly at the Cretaceous–Paleogene boundary^28^. However, these authors did not rule out that the Ir could have derived from an earlier volcanic phase located elsewhere in the CAMP. In the Fundy Basin, sediments less than 1 m below CAMP lavas record several Ir anomalies with values up to 450 ppt (Fig. 2) and were interpreted as volcanic in origin^49^. The Kurusu section of Japan (Fig. 1) is another example of an Ir anomaly associated with the ETE. Here, a highly condensed deep marine section composed of fine-grained chert shows a peak level in Ir (70 ppt), close to a negative C-isotope anomaly that may be equivalent to the the Marshi CIE, based on radiolarians and conodonts (Fig. 2)^31,46^. This Ir anomaly was attributed either to an extraterrestrial bolide impact^31^ or to a volcanic signal^19^. Recently, increased levels of Ir were recorded in the Kuhjoch section. Here, a marked ten-fold increase in Ir from background values <6 ppt to 60–84 ppt coincides with the Marshi CIE and thus with the beginning of the ETE (Fig. 2)^45^. However, it also coincides with a change in lithology from bioclastic wackestone, over a 20 cm thick dark grey, clay- and carbon-rich layer at the base of the Marshi CIE, to more clay-rich sediments above^45^. The highest Ir values occur immediately above the dark grey layer and the Ir values are not correlated to the total organic carbon (TOC) content of the rock, which are low (<0.7%) both below and above the dark grey layer (see Supplementary Fig. 1 showing Ir/TOC%^45^). The Ir values remain high throughout the ETE (41–84 ppt, one outlier at 145 ppt), only decreasing gradually to c. 30 ppt about 6 m above the Triassic-Jurassic boundary^45^.

## Correlation of Pt, Ir, Hg and CIEs associated with ETE

The Ir data are accompanied by data for other PGEs in two (Fundy Basin and Japan)^31,50^, and mercury (Hg) data in three of the sedimentary successions (Morocco, Fundy and Kuhjoch)^7^. To evaluate whether the source of the PGE’s is extraterrestrial or volcanic we will focus here on platinum (Pt), iridium (Ir) and Hg values as shown in Fig. 2, and in the next section also on palladium (Pd) and rhodium (Rh) reported in both datasets^31,50^. First, we note that the sustained Ir anomaly in the Kuhjoch section is accompanied by a similar anomaly in Hg, here reported as Hg/TOC (Fig. 2). However, in the present-day environment Hg is distributed unevenly on a global scale^50^ and linked to atmospheric circulation and wind patterns. Likewise, we expect that the distribution of PGEs as aerosols or particles from a volcanic or impact source also would be governed by atmospheric circulation and wind patterns^51^. Therefore, one should not expect to be able to correlate individual peaks of Ir, Pt or Hg in detail over long-distances. Moreover, this also depends on deposition environment, residence times and sampling density etc. In the Fundy Basin the lowest Ir anomaly occurs 50 cm below the lowest exposed CAMP lava flows and coincides with the base of the CIE whereas a second Ir anomaly occurs c. 20 cm higher up and within the main interval of the Marshi CIE^15,50^. Moreover, the Pt values (up to 1,570 ppt or 1.57 ppb) and Hg show peak levels coinciding with the Ir peaks (Fig. 2). Likewise, in one instance peaks in Ir and Pt correlate positively in the deep marine section of Japan, and may be correlated to the Fundy and Kuhjoch sections as shown in Fig. 2. Although the Ir- and Pt-enriched chert layer in the Japanese section is devoid of volcanic material, volcanic glass fragments were reported from the immediately overlying chert layer^31^. In addition, the uppermost sedimentary layers immediately below (<1 m) the CAMP lava flows in Morocco are enriched in MgO and in mafic sheet-silicates, suggesting that the source-rock of these sediments included eroded, early CAMP basalt^52^. This enrichment in mafic clay minerals and MgO coincides with the base of the CIE (Fig. 2)^15,52^. There are only miniscule anomalies (up to 35 ppt) in Hg in this interval in Morocco (Fig. 2)^7^, consistent with an origin from local erosion of degassed volcanics and basement. The presence of this CIE suggests that the basal lavas in Morocco^9^ likely erupted during the Marshi CIE, providing a temporal link between CAMP volcanism and ETE (Fig. 2). Such a link is further supported by U-Pb geochronological data and by geochemical correlations of magmatism in Morocco^1,5,44^, as well as by palynological investigations of intrabasaltic sedimentary strata^48^.

## PGEs of CAMP basalts from Morocco and ETE sediments

**Figure 3:**
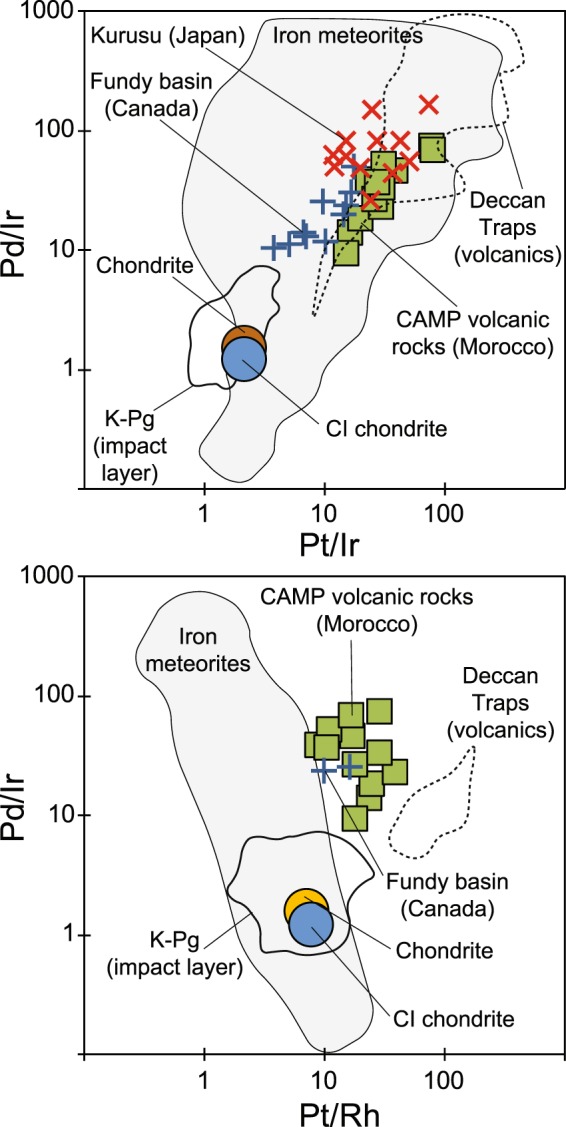
Pd/Ir vs Pt/Ir (**a**) and Pd/Ir vs Pt/Rh (**b**) for volcanic rocks of of this study for the Central Atlantic Magmatic Province (CAMP) in Morocco (see Methods). Also shown are compositions of: (*i*) sediments that coincide with the end-Triassic mass extinction (ETE) in the continental section of the Fundy basin (Passaic formation^50^); a deep-marine section from Kurusu, Japan^31^; (*ii*) CI and ordinary chondrite^54,55^; (*iii*) a field for sediments from the impact layer at the Cretaceous-Paleogene boundary (K/Pg) (field encompassing 106 analyses covering world-wide locations^43^); (*iv*) a field for published compositions of the basalts of the Deccan Traps^56–58^ and (*v*) fields for iron meteorite impactors^64–66^. The published data for the CAMP basalts and ETE sediments are listed in supplementary dataset 2. We have filtered the dataset by Goderis *et al*.^43^ to avoid two sections very close to the Chicxulub impact crater, two sections with anomalous Pt values, and three samples where replicate analyses of Ir deviated by more than 100%.

We analysed PGE concentrations in the CAMP flood basalts from the Argana and the High Atlas basins of Morocco (Fig. 1)^53^. The Ir contents of whole rocks range from 102 to 430 ppt, the Pt contents from 2,638 to 11,319 ppt, and the Pd contents from 2,331 to 10,129 ppt. These values are generally much higher than in the end-Triassic sedimentary sections, but overlap with the values reported in the Fundy basin (Fig. 2)^50^. A key observation is that Pt and Pd concentrations are distinctly enriched relative to Ir in the volcanic rocks as compared to chondrite compositions. This is illustrated in Fig. 3a by elevated Pt/Ir (15–80) and elevated Pd/Ir (9–73) for the CAMP basalts compared to chondrite (Pt/Ir = 2.2; Pd/Ir = 1.2^54,55^). The sediments from Fundy and Japan are similarly enriched in Pt/Ir and Pd/Ir (including the Ir peak levels and surrounding sediments reported by Tanner and Kyte^50^ and Hori *et al*.^31^) (Fig. 3a).

For comparison, Fig. 3a also shows the field for 106 analyses from 34 localities of the well-known impact layer at the Cretaceous-Paleogene boundary^43^. None of these analyses overlap with the sediments associated with ETE, nor with the compositions reported for the coeval flood basalts of the Deccan Traps in India^56–58^, but instead plot relatively close to the chondrite value as expected for impact ejecta^43,59^. However, in particular the Pd/Ir of the Cretaceous-Paleogene boundary layer tend to stretch up to values several times higher than chondrite (Fig. 3a). Goderis *et al*.^43^ explained the spread of sub-chondritic and super-chondritic Pd/Ir values as a result mainly of post-depositional remobilization of Pd, in addition to possible primary fractionation during condensation of the impact vapor plume. The post-depositional remobilisation is consistent with Pd being more mobile than Pt that, in turn, is more mobile than Ir^60–63^. Similar to the Pd/Ir of the Cretaceous-Paleogene boundary, the Pd/Ir of the ETE sediments also appears to be shifted towards slightly higher values relative to the CAMP volcanic rocks (Fig. 3a). By analogy, we explain this as the result of post-depositional remobilisation of Pd.

Finally, it is appropriate also to discuss the possibility of an iron meteorite impactor as the source for the PGEs in the ETE sediments. Indeed, in Pd/Ir vs Pt/Ir space the known iron meteorites^64–66^ cover a broad field that overlaps with the ETE sediments (Fig. 3a). However, iron meteorites are known to be relatively enriched in rhodium (Rh), resulting in low Pt/Rh values^64–66^. In Fig. 3b, it is clear that the two sediment samples with Rh values above detection limit have Pt/Rh values that are higher than known iron meteorites and, again, are comparable to the compositions of the CAMP basalts. We therefore conclude that the elevated PGE anomalies of ETE sections are best explained by a volcanic source and cannot be explained by chondritic or iron impactors. However, further high-precision PGE data (that include Rh) for the sedimentary successions are needed to substantiate this conclusion.

## Discussion

The fractionated nature of the PGEs (e.g. high Pt/Ir and high Pt/Rh) of ETE sediments demonstrates that Ir, Pt and Pd anomalies associated with ETE are inconsistent with chondritic or iron impactors, and are best explained as volcanic in origin (Fig. 3). This is consistent with several independent observations such as multiple levels with elevated Ir concentrations, lack of shocked minerals or spherules in the sediments, and lack of an impact structure of appropriate age and size^33,38,45,49,50^. This is also consistent with elevated Hg (and Hg/TOC) values reported for ETE sediments that have been interpreted as volcanic in origin^7,8,27^ (Fig. 2). The coincidence of Ir, Pt and Hg anomalies with negative CIEs and ETE (Fig. 2) therefore demonstrates that global, paleo environmental perturbations at the Triassic-Jurassic boundary took place exactly at the time when there was an unusual PGE flux that was fractionated relative to chondrite or bulk mantle. For example, at Kuhjoch the Ir anomaly starts precisely at the Marshi CIE (i.e. at the beginning of the extinction event) and ends shortly into the Jurassic period^45^, spanning the duration of the main phase of CAMP volcanism. If the PGE flux is linked to CAMP, then volcanism must have started slightly before the emplacement of the preserved lava flows in the Fundy basin (and possibly in the Newark basin), corroborating the radiogenic chronology showing that CAMP intrusions predate the known erupted lavas^5,6,12^. This is consistent with the hypothesis that CAMP volcanism in Morocco predates the North American sector of the province^12,15,48,52,67^. The few data sets available so far (Fig. 2) suggests the occurrences of excess Ir and other PGE anomalies in the ETE sediments are found both close to, and far away from CAMP (Fig. 1). This suggests that these elements were distributed globally and this is best explained by loading of PGEs to the atmosphere as aerosols^68,69^, particles^70^ or compounds complexing with chlorine^68^ by CAMP volcanism. Similarly, Rampino *et al*.^71^ has recently argued that excess nickel anomalies in sediments of the end-Permian extinction were best explained as atmospheric loading from the eruption of the Siberian Trap basalts. In addition and more locally, erosion of the vast surface area (>10 million km^2^)^7^ of Pangaea covered by CAMP basalts (Fig. 1) likely also contributed to excess PGE anomalies, e.g in the continental basins such as observed in the Fundy basin (Fig. 2). If as little as 0.3% of the total Ir in the magma escaped to the atmosphere as measured in plumes over Hawaiian volcanoes^72^, we estimate that CAMP may have released c. 6 million kg Ir and 157 million kg Pt to the atmosphere, together with an abundance of other deleterious gases^6–8,73^. Hence, we propose that PGEs can provide a robust method for tracing volcanic events (as well as impacts) in marine and continental environments. For the end-Triassic mass extinction, any significant contribution from an asteroid impactor can be ruled out.

## Methods

This paper combines platinum-group element (PGE) and other data from the literature to test the hypotheses that excess Ir and other PGE anomalies in sedimentary successions of the end-Triassic mass extinction are related either to volcanism of the Central Atlantic Magmatic Province (CAMP) or to extraterrestrial impact. The present paper is a companion to a recent Journal of Petrology contribution that reported and described PGE data of CAMP in detail and focused on modelling and constraining mantle melting dynamics^53^. Twelve samples of CAMP flood basalt from Morocco were selected for PGE analysis and are representative of a larger sample set. Care was taken during sampling to select samples as fresh and unweathered as possible. Moreover, weathered surfaces and alteration veins were cut away before analyses. The least altered samples were selected for this study based on petrographic inspection and low loss-on-ignition (LOI) measured on whole rock powder (0.9 to 3.3 wt%, with an average of 1.9 wt%, Supplementary Datafile 1)^53^. Moreover, with exception of Os, the PGEs are not mobile during typical surface weathering^74^. We therefore assume, similar to many other studies^75,76^, that the measured whole rock PGE compositions of fine grained lavas represent the concentrations in the original magma. The whole rock concentrations of PGE were analysed using nickel sulphide fire assay pre-concentration and tellurium co-precipitation followed by inductively coupled plasma-mass spectrometry at Cardiff University^77,78^, as detailed in ref. ^53^. The certified reference materials WITS1, TDB1, and WPR1 were analysed together with the unknowns (Supplementary Datafile 2). Most values fall within the standard error of the reference material, while this does not apply for Ir, Pd and Au in TDB1. The values for these three elements in TDB1 fall between 1–2 standard errors of the certified values but Ir, Rh and Pd are in better agreement with the recommended compilation values from Meisel and Moser^79^ and the majority of recent values for Ir, Rh and Pd in TDB1 reported in the GeoREM database^80^. The measured PGE and Au concentrations are at least 10 times larger than the minimum detection limit for the method^78^. Duplicate analyses of three samples generally deviate less 13%, apart from Rh deviating 22% and 32% in two samples, respectively, and Os deviating 22% in one sample.

The data for CAMP basalts^53^ and for the sedimentary successions^31,50^ are listed in Supplementary Datafile 1 for easy reference.

## Supplementary information

Supplementary Dataset 1.
